# Development of the Online Money Management Credit Card Task

**DOI:** 10.1093/geroni/igaa057.2906

**Published:** 2020-12-16

**Authors:** Preeti Sunderaraman, Ziqian Dong, Santhoshkumar Sampath, Silvia Chapman, Jillian Joyce, Yaakov Stern, Stephanie Cosentino

**Affiliations:** 1 Columbia University, New York, New York, United States; 2 New York Institute of Technology, New York, New York, United States; 3 Columbia University, New York, Pennsylvania, United States; 4 Columbia University Medical Center, New York, New York, United States

## Abstract

Older adults (OAs), a wealthy but vulnerable segment of our population, are at risk to make compromised financial decisions. Evidence suggests that OAs increasingly use technology to perform everyday financial transactions, such as to manage their credit card statements. However, current tools are lacking in terms of assessing how older adults navigate and handle the online financial milieu. We will discuss the development of a novel, simulated online money management (OMM) credit card statement task. OMM examines OAs performance on several indices including reaction time, nature and frequency of errors, and their ability to comprehend and trouble shoot problems. Psychometric properties related to the reliability and validity will be discussed. Ultimately, by examining the longitudinal performance of OMM in OAs, we can better characterize the natural course of OMM. Such an approach will enable clinicians to accurately and objectively examine OMM and identify those at risk for making financial errors.

